# Iron Transport Tocopheryl Polyethylene Glycol Succinate in Animal Health and Diseases

**DOI:** 10.3390/molecules24234289

**Published:** 2019-11-25

**Authors:** Ajay Srivastava, Rajiv Lall, Jamil Talukder, Dan DuBourdieu, Ramesh C. Gupta

**Affiliations:** 1 Vets Plus, Inc., Menomonie, WI 54751, USA; drajay@vets-plus.com (A.S.); lallr@vets-plus.com (R.L.); jamilt@vets-plus.com (J.T.); dand@vets-plus.com (D.D.); 2 Breathitt Veterinary Center, Toxicology Department, Murray State University, Hopkinsville, KY 42240, USA

**Keywords:** ITPGS, bioenhancer, solubilizer, stabilizer, bioavailability, phytomedicine, nanomedicine, drug delivery, antioxidative, anti-inflammatory, immunomodulatory

## Abstract

Gut health is the starting place for maintaining the overall health of an animal. Strategies to maintain gut health are, thus, an important part in achieving the goal of improving animal health. A new strategy to do this involves two molecules: the iron transport protein ovotransferrin (IT) and α-tocopheryl polyethylene glycol succinate (TPGS), which result in the novel formulation of ITPGS. These molecules help reduce gut pathogens, while enhancing the absorption and bioavailability of therapeutic drugs, phytomedicines, and nanomedicines. This, in turn, helps to maintain normal health in animals. Maintaining the gastrointestinal tract (GIT) in its normal condition is key for successful absorption and efficacy of any nutrient. A compromised GIT, due to an imbalance (dysbiosis) in the GIT microbiome, can lead to an impaired GI barrier system with impaired absorption and overall health of the animal. The molecules in ITPGS may address the issue of poor absorption by keeping the GI system healthy by maintaining the normal microbiome and improving the absorption of nutrients through multiple mechanisms involving antioxidative, anti-inflammatory, immunomodulatory, and antimicrobial activities. The ITPGS technology can allow the dose of active pharmaceutical or herbal medicine to be significantly reduced in order to attain equal or better efficacy. With complimentary actions between IT and TPGS, ITPGS presents a novel approach to increase the bioavailability of drugs, phytoconstituents, nutrients, and nanomedicines by enhanced transport to the tissues at the site of action, while reducing gut pathogen load. The ITPGS approach appears to be a novel strategy for maintaining the health of animals by manipulation of microbiota.

## 1. Introduction

Intestinal health is critically important for the welfare and performance of animals and humans. Enteric diseases that affect the structural integrity of the gastrointestinal tract (GIT) lead to high economic losses due to reduced weight gain, poor feed conversion efficiency, increased mortality rates, and greater medication costs. The overall health of an animal typically starts in the gut. This is due to two key aspects of the gut: it is the site in the body where an animal absorbs nutrients, but also where pathogens are usually encountered first. Researchers have, thus, focused efforts on identifying methods to maintain overall health in animals. The use of nutraceuticals is one such method. In in vivo studies, many nutraceuticals were reported to be poorly absorbed in the GIT, thus limiting bioavailability [1,2,3,4,5,6,7]. The dissolution and ability to permeate the GI wall are considered to be major barriers to drug/phytomedicine bioavailability [6,8]. As a result, the concentration of bioactive phytoconstituents(s) at the target site(s) is very low, thereby having a minimal effect. In addition to low bioavailability, natural products have poor solubility and stability. To overcome these obstacles, bioenhancers of herbal and non-herbal origins are indicated for drugs and phytochemicals [9,10,11]. The use of bioenhancers and potentiators dates back thousands of years [12,13,14]. Piperine from black pepper appears to be the first example of Yogvahi (herbs that are able to increase or potentiate the plasma concentration of drugs) mentioned in Ayurvedic medicine [15,16,17,18]. In other in vivo studies, peppermint oil has been reported to increase bioavailability of cyclosporine [19], and ginseng polysaccharides have been reported to enhance absorption of ginsenoside Rb1 [20].

In in vivo studies, Yurdakok-Dikmen et al. [11] reported that following oral administration, the biodegradation of herbal drugs occurs in the stomach or gut lumen, where membrane permeability and metabolism are key factors in absorption. Bioenhancers are from various classes and exert their effects via multiple mechanisms, such as enzymatic alterations, phytosome formulations, transporter protein alterations, cholagogue/cholerectic effect, and thermogenesis. Absorption of drugs/phytoconstituents can be enhanced by employing various strategies, such as manipulating formulation (micronization, nanocrystals/nanosuspensions, solid solutions, self-dispersing solid solutions with surfactants, lipid solutions, self-emulsifying drug delivery systems, and surfactant-cosolvent systems) [11,21].

In a number of in vivo studies, properties of TPGS (vitamin E TPGS) have been described to enhance the delivery of drugs, nutraceuticals, nanoparticles, and dietary nutrients [6,22]. The poor water solubility of vitamin E limits its absorption in the GIT, but esterification of vitamin E succinate with polyethylene glycol (PEG) 1000 makes it completely water soluble [6,23,24,25]. TPGS has an amphiphilic structure comprising of a hydrophilic polar head portion and lipophilic alkyl tail. It can be functionalized as an excellent solubilizer, emulsifier, and permeation and bioavailability enhancer of hydrophobic drugs and nutraceuticals [6,26,27,28,29]. TPGS can also be used in the development of nanomedicines, focusing on simple structures with multifunctional properties [6,29,30]. Of course, the preparation of nanomedicines is still at the laboratory scale and progress on developing novel nanomedicines is relatively slow. In both in vitro and in vivo studies, TPGS has also been shown to exhibit additional properties, such as a surfactant, apoptogenic, anticancer, immunomodulator, neuroprotection, and reversal of multidrug resistance (MDR) by inhibiting P-glycoprotein (P-gp) activity [6,29,31,32,33,34,35,36]. TPGS 1000 (1000 denoting the PEG chain molecular weight) has been approved by the FDA as a GRAS supplement to solubilize drugs in oral, parenteral, topical, nasal, and rectal/vaginal therapies [31,37,38,39]. TPGS has also been approved by the European Food Safety Authority (EFSA) as a safe pharmaceutical adjuvant used in drug formulation and for research purposes [39,40,41].

Vitamin E TPGS is a powerful tool in the formulation of poorly-soluble lipophilic compounds, as it enhances absorption and bioavailability via efficient micellar uptake into enterocytes. TPGS has a proven record of safety and efficacy in pharmaceutical, dietary supplement, food and beverage, personal care, animal nutrition, and cannabinoid products. It has the added benefit of being a clean label ingredient. In order to achieve greater stability, bioavailability, additional biological/pharmacological effects, and better therapeutic efficacy, TPGS technology has been further improvised by combining the iron transport (IT) protein of ovotransferrin with TPGS. The formulation of Iron Transport Polyethylene Glycol Succinate (ITPGS) is soluble in both fat and water and exerts multiple biological and pharmacological effects, such as antioxidative, anti-inflammatory, antimicrobial, immunomodulatory, healthy gut, and overall health improvement. This review describes various facets of ITPGS, including formulation, biological properties, pharmacological mechanisms, and therapeutic efficacy.

## 2. Source of Iron Transport Polyethylene Glycol Succinate (ITPGS) and its Formulations

**Figure 1:**
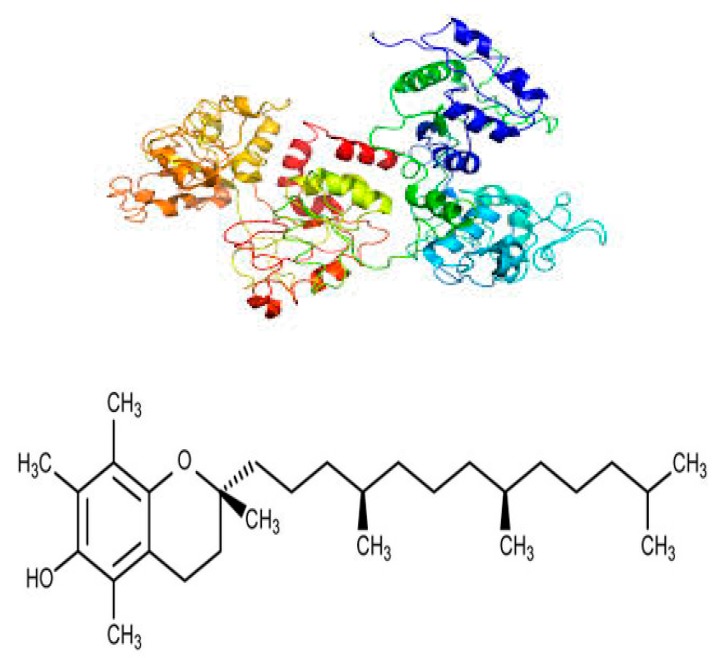
Structural formula of ITPGS (IT.TPGS).

d-α-Tocopheryl polyethylene glycol succinate (TPGS) is prepared from natural lipid-soluble α-tocopherol (vitamin E) by grafting to a polyethylene glycol (PEG) oligomer through a succinate diester linker [30]. The iron transport polyethylene glycol succinate (ITPGS) molecule, in addition to natural α-tocopherol, incorporates the iron transport aspect from ovotransferrin (formerly known as conalbumin) obtained from egg-white or its hydrolysates [42,43,44,45]. The structural formula of ITPGS (IT.TPGS) is shown in Figure 1.

## 3. Uses of ITPGS

Tocopheryl polyethylene glycol succinate (TPGS) has been used for many purposes, including as an efficient source of natural vitamin E, both for therapeutic and nutrition purposes. Vitamin E TPGS (a water-soluble derivative of vitamin E) is a non-ionic surfactant used as a solubilizer, absorption enhancer, emulsifier, vehicle for lipid-based drug/herbal formulation, and antioxidant. ITPGS, by containing iron from ovotransferrin, offers the possibility of vastly improved effects for maintaining normal health, especially in exerting antimicrobial effects in the gut, where pathogens are typically encountered first in an animal. In in vitro and in vivo studies, ovotransferrin has been reported to be involved in iron transport and iron delivery, and has antibacterial [46,47,48,49], immunomodulatory [44,50,51], and ROS scavenging activities [44,50,51]. By having α-tocopherol and iron transport, ITPGS appears to be a unique and novel composition of two moieties with a wide range of biological and pharmacological activities.

## 4. ITPGS as a Bioenhancer

In the past, a number of drug delivery systems, including liposomal formulations, nanoparticles (nanocrystals and nanosuspensions), and micellar formulations, have been developed and investigated for enhanced absorption and bioavailability. ITPGS/TPGS may not only increase the bioavailability of pharmaceuticals, herbal products, and nanomedicines, but also of nutrients, which are otherwise poorly soluble and less bioavailable. ITPGS has the potential to maintain a healthy gut environment for normal enterocytes, by improving uptake, transport, and bioavailability of nutrients. The mechanism by which water-soluble TPGS enters the enterocytes and becomes absorbed has been described by Neophytou and Constantinou [25]. The TPGS portion of ITPGS is hydrolyzed to free α-tocopherol in the stomach by nonenzymatic hydrolysis, in the proximity of the brush border epithelium by esterase hydrolysis, and on the surface of enterocytes via a lipase. As such, micelles of ITPGS with other substances can potentially pass through cell membranes, thereby enabling the absorption of the ITPGS, as observed with TPGS [6,37,52].

In vivo and in vitro studies suggest that the absorption, bioavailability, intracellular uptake, and localization of α-tocopherol is greater than any other natural isoforms of vitamin E [53,54,55,56,57,58,59,60]. Following oral administration of α-tocopherol and α-tocotrienol in mice, Khanna et al. [61] found detectable levels of both isoforms in the skin, heart, lungs, brain, liver, bone marrow, and blood, suggesting that these isoforms of vitamin E can be effectively transported to various organs and that they display nonspecific distribution to tissues. In in vivo studies, uptake and distribution of vitamin E have been shown to be increased by making it water soluble, such as in the TPGS or ITPGS form.

In in vivo and in vitro studies, water-soluble vitamin E TPGS has been investigated for various properties, such as solubilizer, micellar property as a surfactant, additive or emulsifier in drug formulations and in drug delivery systems, P-gp inhibitor, oral absorption enhancer, permeation enhancer, and bioavailability enhancer [6,27,30,32,62,63]. The ATP-binding cassette superfamily contains membrane transporter proteins that transport a wide range of diverse compounds across cellular membranes. P-gp is an important member of this family and a multi-specific drug efflux transporter that plays a significant role in governing the bioavailability of many clinically active drugs [64]. P-gp is primarily thought to serve as a natural protective function, by transporting substances, including toxins, from bacteria [65] out of cells. P-gp of epithelial cells is a membrane protein located in the apical membrane and functions as a multidrug efflux pump [66]. TPGS acts as a reversal agent for P-gp mediated MDR and inhibits P-gp mediated drug transport. These findings suggest that enhanced oral bioavailability of drugs co-administered with TPGS may, in part, be due to inhibition of P-gp in the intestine. As observed with TPGS, co-administration of ITPGS may enhance drug solubility, enhance P-gp mediated MDR, and increase the oral bioavailability and cellular uptake of drugs, nanoparticles, and herbal medicines. Collnot et al. [32] investigated TPGS analogs that varied in their PEG chain length, and/or possessed a modified hydrophobic core. Transport studies revealed that modulation of ATPase activity correlated with inhibitory potential for P-gp mediated efflux. These investigators suggested that ATPase inhibition is an essential factor in the inhibitory mechanism of TPGS on cellular efflux pumps [32]. A mechanistic understanding of the inhibition of P-gp by TPGS gives new insights into the structure activity relationship and improves rational design of more potent P-gp inhibitors.

In an in vivo study, Chen et al. [2] demonstrated that TPGS significantly enhanced oral bioavailability of the phytomedicine berberine chloride (BBR): peak concentration (C*_max_*) increased by 2.9 times and area under the curve (AUC_0–36_) increased by 1.9 times in rats. It has been reported that BBR is a substrate of P-gp, and that P-gp plays an important role in the absorption of BBR [2,67]. The absorption enhancing property of TPGS may be due to its ability to affect the biological activity of P-gp and thereby reduce the excretion of absorbed BBR into the intestinal lumen. Currently, TPGS is considered a proven bioenhancer of BBR. TPGS has also been reported to enhance absorption and bioavailability of several drugs of different classes [23,39,68,69,70,71,72,73]. In a number of in vivo studies, TPGS has also been shown to enhance absorption and bioavailability of nutraceuticals [2,6,74,75,76]. It is hypothesized that ITPGS is expected to be a better bioenhancer than TPGS because with potentially less pathogenic bacteria present, less energy is required by enterocytes and the gut to fight pathogens.

## 5. Free Radical Scavenging and Antioxidative Properties

The antioxidative properties of ITPGS are based on their hydrolysis by cytoplasmic esterases that liberate free α-tocopherol, which then localizes in the cell membranes and quenches free radicals. This protects the membrane from lipid peroxidation and damage [30,77,78]. ITPGS works as a free radical scavenger and antioxidant in stressed cells and thereby promotes a healthy environment. Both TPGS and ovotransferrin molecules have shown antioxidant properties via different mechanisms. In both in vitro and in vivo studies, isoforms of natural vitamin E have been reported to exert various biological and pharmacological properties, such as antioxidative, anti-inflammatory, neuroprotective, antithrombotic, and anticancer [25,77,78,79,80].

Previous in vitro and in vivo studies have shown that mitochondria contain the highest concentration of vitamin E and its analogs and that vitamin E accelerates ATP resynthesis [81,82,83]. Vitamin E mainly acts as a chain-breaking antioxidant and radical scavenger, protecting cell membranes against oxidative damage [81,84,85,86]. In addition, vitamin E regulates ROS production [86], maintains oxidative phosphorylation in mitochondria, and accelerates restitution of high-energy phosphates [87,88]. In in vitro studies, mitochondrial targeting α-tocopherol and α-tocopheryl succinate have been shown to exert apoptotic and anticancer properties [83,89,90,91].

From in vitro and in vivo studies, it is well established that α-tocopherol and other isoforms of vitamin E, in addition to antioxidant properties, can interact with enzymes, proteins, lipids, and transcription factors [25,92]. α-Tocopherol can inhibit the function of PKC, 5-lipoxygenase, and phospholipase at the posttranscriptional level and can activate phosphatase 2A. α-Tocopherol is also reported to inhibit cellular proliferation, platelet aggregation, and monocyte attachment [93]. An antioxidative effect can be attributed to ovotransferrin [44] as well as α-tocopherol. Both in vitro and in vivo studies have also shown that ovotransferrin can enhance the antioxidative effect of other nutraceutical ingredients. For example, conjugation of ovotransferrin with catechin showed improved antioxidant activity [94].

## 6. Anti-Inflammatory Properties

Gastrointestinal inflammation can be induced by a large number of microorganisms [95,96,97,98,99]. The microbial imbalance (dysbiosis) can be caused by several factors, such as diet, disease, infection, medications, toxicants, aging, etc. A dysbiotic microbiota may be sufficient to provoke intestinal inflammation, such as that seen in inflammatory bowel disease, and there is much current interest in quantitative and/or qualitative abnormalities of the microbiota that may be associated with systemic immune, allergic, obesity, metabolic, and infectious disorders, periodontal and respiratory diseases, and cancer [99,100,101,102,103,104,105]. In a number of in vivo and in vitro studies, the interaction between the gut microbiota and the immune system has been investigated [99,106,107,108]. Nieuwenhuizen et al. [109] hypothesized that failure of the gut is an important pathophysiological phenomenon of the generalized inflammatory response that leads to multiple organ dysfunction syndrome (MODS). Abnormal colonization, infection of gut origin, and bacterial translocation are all signs of gut failure that have been implicated in the pathogenesis of MODS.

**Figure 2:**
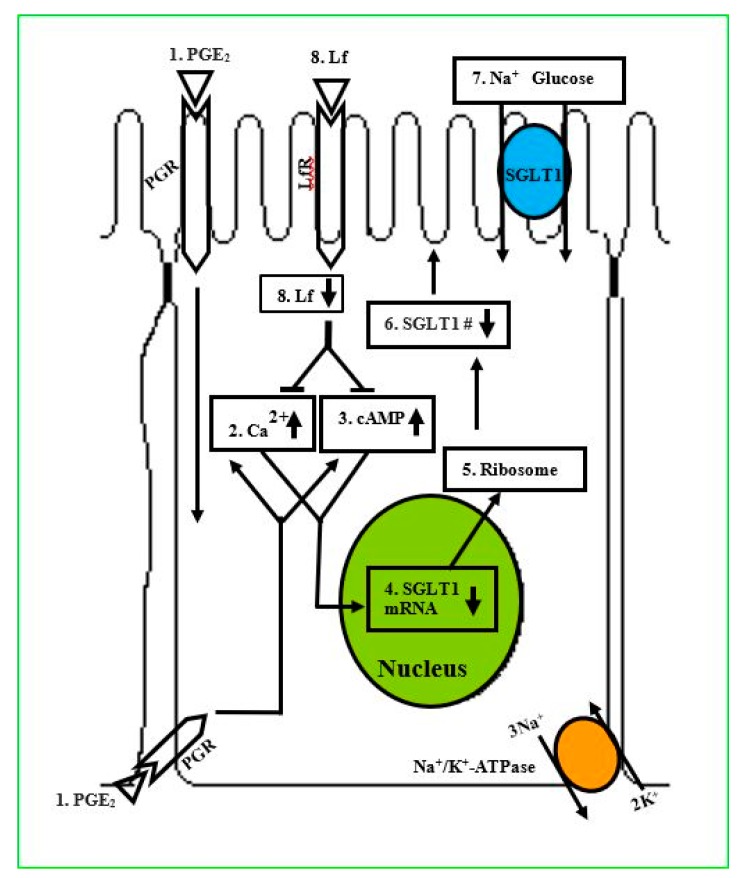
Lactoferrin restoration of glucose absorption.

In an in vitro study, Talukder et al. [110] examined the actions of lactoferrin (Lf) to prevent the prostaglandin E_2_ (PGE_2_) effect on Na^+^-dependent glucose cotransport (SGLT1) for glucose absorption in enterocytes. These investigators grew intestinal epithelial cells (IEC-6) on transwell plates, treated with phlorizin, PGE_2_, AH6809, and Lf, and performed 3-*O*-methyl d-glucopyranose (OMG) uptake within 10 days postconfluent. Na^+^-dependent OMG uptake, phlorizin, and immunoblotting studies showed the activity and apical membrane localization of SGLT1 in IEC-6 cells. Findings revealed that PGE_2_ inhibits SGLT1 in a concentration- and time-dependent manner with an inhibitory constant (Ki) of 50.0 nmol/L and was antagonized by prostanoid receptor inhibitor, AH6809. It was also observed that PGE_2_ did not alter Na^+^/K^+^-ATPase activity. In contrast, quantitative real-time polymerase chain reaction (qRT-PCR) and Western blot analysis revealed that SGLT1-specific transcripts and protein expression level were decreased 3-fold by PGE_2_. Furthermore, PGE_2_ treatment increased intracellular cAMP and Ca^2+^ concentrations and decreased SGLT1 expression on the apical membrane, and these effects were ameliorated by Lf. It was concluded that Lf ameliorates the PGE_2_ inhibition of SGLT1 via the Ca^2+^- and cAMP-signaling pathways (Figure 2). Taken together, iron binding protein molecules such as Lf or ovotransferrin appear to play an important role in significantly reducing inflammation of the intestine and restoring the absorption of nutrients.

Figure 2 shows a schematic pathway for glucose absorption in cells of a PGE_2_-mediated inhibition of SGLT1 that is intercepted by Lactoferrin (Lf). (1) PGE_2_: extracellular PGE_2_. PGR: Prostanoid receptors localized on the plasma membrane. (2) Ca^2+^1: increased intracellular Ca^2+^ concentration level ([Ca^2+^]i) because of PGE_2_. (3) cAMP: increased intracellular cyclic adenosine Monophosphate ([cAMP]i) level because of PGE_2_. (4) SGLT1 mRNA: SGLT1 mRNA transcription and abundance are decreased because of PGE_2_. (5) Ribosome: synthesis of SGLT1 protein numbers depend on the abundance of SGLT1 mRNA transcripts. (6) SGLT1 protein no.: quantity of SGLT1 protein on the apical membrane is reduced. (7) Na^+^-Glucose: Na^+^-dependent glucose uptake is reduced because of PGE_2_. LfR: Lf receptors. 8) Lf: Lactoferrin endocytosized by receptor-mediated transcytosis and inhibits the PGE_2_-mediated upregulation of [Ca^2+^]i and [cAMP]i levels, and increased Na^+^-glucose uptake in dependent IEC-6 cells by increasing the Vmax (courtesy of Talukder et al. [110]). It is reasonable to believe that ovotransferrin may also work in the same manner and have a similar mechanism to increase glucose uptake.

ITPGS works as an inflammatory regulator or balancer to maintain normal gut health. Iron is greatly required by pathogenic bacteria in metabolic processes for their growth and replication, in contrast to probiotics (non-pathogenic bacteria) that do not need much iron. The IT molecule sequesters iron and deprives pathogenic bacteria from iron. In addition, IT molecules cause osmolysis. Thus, IT molecules can act as a bacteriostatic or bactericide for pathogenic bacteria, while they do not kill probiotics in the gut. Furthermore, ITPGS appears to maintain a healthy inflammatory response in the body by regulating biomarkers such as interleukins and TNF-alpha. The anti-inflammatory property of ITPGS can also be attributed to vitamin E [78].

## 7. Antimicrobial Properties

In vitro and in vivo studies suggest that the iron (Fe^2+^ or Fe^3+^)-binding protein ‘transferrin’ in ovotransferrin (from egg-white) or lactoferrin (from colostrum and milk) exerts antimicrobial [111,112,113,114,115,116,117,118], immunomodulatory [117], anti-inflammatory [119] properties, and anti-cancer [120] effects. In an in vitro study, Valenti et al. [111] reported that *Pseudomonas* spp., *Escherichia coli*, and *Streptococcus mutans* were the most sensitive and *Proteus* spp. and *Klebsiella* spp. were the most resistant to the antibacterial action of ovotransferrin.

IT molecules starve pathogenic bacteria (Gram positive and Gram negative) of iron, and the same IT molecules greatly enhance the probiotic bacteria (*Lactobacillus* and *Bifidobacteria*) [118]. In addition, endometritis, usually caused by an infection in 7–20% of dairy cows within 10 days postpartum, deteriorates health, decreases milk production, and causes a huge economic loss to farms. Unpublished in vivo studies with IT molecules showed that rectal temperature returned to normal after one week of intrauterine ovotransferrin treatment. Mean pH of uterine discharge from metritis cows was 8.8 (control 6.8) and changed to 7.0 after one week of ovotransferrin treatment. Mucus score was 6 times higher, and total number of polymorphs was more than 70 times higher in cows with metritis and significantly (*p* < 0.01) reduced (2 and 4 times, respectively, compared with control) after ovotransferrin treatment. Bacterial culture of uterine swabs demonstrated the presence of different types of bacteria, including Gram positive, Gram negative, α- and β-hemolytic, rods, and cocci. Zone of inhibition studies with different concentrations of ovotransferrin demonstrated that 5% would be more than enough to kill all types of bacteria found in these studies. Thus, it was concluded that IT molecules are a potent antimicrobial protein and have the ability to kill different types of bacteria in the gut and especially pathogenic bacteria that require iron in in vitro and clinical applications. While numerous pathogenic bacteria need iron to survive, many probiotic spp. (such as *Lactobacillus* spp.) need less iron [121] or no iron [122,123] for their survival. Indeed, orally administered iron has a direct impact on microbial composition in the gut. It can result in reduction in the beneficial microbiota and the expansion of pathobionts, and this can also provide an opportunity for the expansion of enteric pathogens [124]. As such, the concept of limiting or having just enough iron in the gut may have some beneficial aspects for general health of an animal. ITPGS promotes a healthy gut environment for normal microbial growth. Thus, nutrients can be more readily absorbed due to a reduction of pathogenic bacteria that may be competing for nutrients.

In the gut, ITPGS molecules are absorbed, and then exert their biological and pharmacological actions. Ovotransferrin deprives pathogenic bacteria of iron and other nutrients needed for survival. Keep in mind that TPGS inhibits P-gp, and it is necessary to reduce pathogens that may be producing toxins that would normally be removed from enterocytes by P-gp. The combination of TPGS with ovotransferrin allows this to occur. Less pathogenic bacteria present in the GIT may promote the growth of probiotic bacteria, thereby creating a healthy microbial balance. In addition, IT molecules improve the health of intestinal villi by scavenging free radicals and reducing oxidative stress, thus improving the healthy inflammatory response.

## 8. Effects on Gut Microbiota and Enterocytes

The relevance of gut microbiome and microbiota is well established in health and diseases of humans and animals [99,125,126]. In healthy gut, a microbial ecosystem exists in eubiosis state characterized by a dominance of potentially beneficial microbial species. However, dysbiosis is an imbalance in composition and number of gut commensal microorganisms characterized by a heavy load of pathogenic bacteria [127] and altered gut microbiome [128]. Emerging evidence suggests that dysbiosis may adversely influence the intestinal microbiome and eventually GI structure and function. Indiscriminate use of antibiotics, chemotherapy, and stress have been implicated in the development of dysbiosis. The host’s iron and dietary iron are reported to alter the microbiota composition leading to dysbiosis [128]. In a recent study, the effect of ovotransferrin was investigated on cyclophosphamide-induced intestinal microbiota and dysbiosis [129]. The study suggested that ovotransferrin improved the diversity and richness of the intestinal microbiome, and therefore ITPGS is likely to reverse dysbiosis to eubiosis.

Currently, a large number of in vivo studies suggest that nutraceuticals (including prebiotics, probiotics, and synbiotics) are used to maintain the normal gut biota and to prevent or treat GIT-related diseases [117,118,130,131,132,133,134,135,136,137,138]. The GIT contains a complex polymicrobial community that consists of bacteria, viruses, archaea, fungi, and parasites [99,139,140,141]. The bacterial community is the most prominent and important factor in maintaining homeostasis of this complex gut environment. The human gut microbiota is composed of more than one hundred trillion microbial cells, including aerobic and anaerobic species as well as Gram-positive and Gram-negative species [142]. The term ‘microbiota’ is defined as a set of commensal, autochthonic microorganisms, co-existing with a host without causing any harm [143,144]. Freter [145] defined intestinal microbiota as “the usually complex mixture of bacterial populations that colonize a given area of the GIT in individual human or animal hosts that have not been affected by medical or experimental intervention or disease”. Gut microbiota differ substantially among animal strains and species, gender, and between humans and animals [99,135,140,141,146,147,148].

It is well established that microbiota plays a great role in the health of the GIT as well as of other vital organs [149,150,151,152,153]. The GIT is the largest reservoir of commensal bacteria in the human or animal body, providing nutrients and space for the survival of microbes while concurrently operating mucosal barriers to confine the microbial population [154,155]. Commensal organisms are known to promote gut health through the induction of mucus production and enterocyte turnover [156]. As such, the gut microbiota is a key factor in shaping the biochemical profile of the diet and, therefore, its impact on host health and disease [141,157,158,159,160]. In an in vitro study, Yu et al. [155] also described that the epithelial cells linked by tight junctions not only physically separate the microbiota from the lamina propria, but also secrete proinflammatory cytokines and reactive oxygen species (ROS) in response to pathogen invasion and metabolic stress and serve as a sentinel to the underlying immune cells. Commensal bacteria are involved in various physiological functions in the gut, and inflammation and microbial imbalance (dysbiosis) may cause bacterial invasion and pathology [140,155,161,162,163,164,165].

In in vitro and in vivo studies, Rowland et al. [160] described that gut microbiota-derived products can be absorbed from the gut and enter the host’s endogenous and exogenous pathways to influence the overall metabolic phenotype of the host. In addition, metabolites generated by the host can be secreted into the gut via the enterohepatic circulation, and serve as substrates for the resident microbes. It is evident from in vivo and in vitro studies that gut microbiota not only influence the metabolism, health, and diseases of GIT, but also of other vital organs, including liver, kidney, nervous system, and immune system [99,140,142,153,155,158,166,167,168,169,170,171,172]. Recent in vivo studies investigating interactions between microbial communities and the host have shown that gut bacteria can cause or contribute to the occurrence of conditions such as allergies, inflammatory bowel diseases, rectal cancer, diabetes, obesity, and behavioral alterations [101,103,105,108,141,153,173,174]. In fact, failure of the gut can lead to MODS, as in most cases, gut dysfunction precedes the development of MODS [109,172,175,176]. It is plausible that gut bacteria may be initiating and perpetuating the production of local inflammatory mediators, which may produce distant organ injury [105,109,152,153,172,177,178].

**Figure 3:**
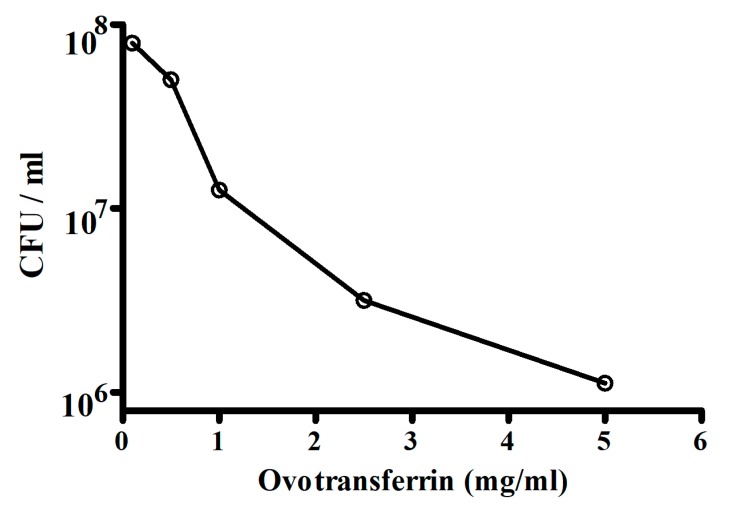
Antibacterial activity of ovotransferrin (OvTf) against E. coli strain W1485 (5 × 10^5^ CFU/mL) of different concentrations of OvTf. Ordinate, colony-forming units per milliliter; abscissa, concentrations of OvTf (courtesy of Valenti et al., 1982 [111]). Antibacterial activity increased with concentrations of OvTf.

*Escherichia coli* is one of the important causes of diarrhea in humans and animals. The antibacterial activity of ovotransferrin towards *Escherichia coli* strain W1485 was tested by Valenti et al. [111]. The bacterial growth was measured in colony-forming units (CFU) and the results of the antibacterial activity of ovotransferrin are shown in Figure 3. The antibacterial activity increased with the concentrations of ovotransferrin. They indicated that in the presence of ovotransferrin, slow changes that progressively limit their ability to grow may occur in the microorganisms. It could be argued therefore that these changes reflect a direct interaction of the protein with the bacteria or a deprivation of an internal iron pool in slow equilibrium with the medium or both.

Lactoferrin is an iron-binding glycoprotein, belonging to the transferrin protein family that ovotransferrin also belongs to. Lactoferrin has antioxidant and antimicrobial properties and has been used for treating diarrhea [179]. Other uses include stimulating the immune system, preventing tissue damage related to aging, promoting healthy intestinal bacteria, preventing cancer, and regulating the way the body processes iron. The iron binding actions of ovotransferrin and lactoferrin make it useful to research for functional similarity and functional substitutions in formulations. For example, in vitro studies Valenti et al. [180] showed that ovotransferrin and lactoferrin have nearly identical abilities for antimicrobial activity against *E. coli* and *Streptococcus mutans*. This suggests functional equivalency between ovotransferrin and lactoferrin for at least their bacteriostatic actions against pathogenic bacteria and provides further rationale for ovotransferrin in formulations that may have relied on lactoferrin.

An example of where ovotransferrin may have equivalency with lactoferrin is in an in vivo study of Talukder and Harada [181], who investigated the effects of the iron-binding multifunctional glycoprotein, bovine lactoferrin (BLf), on lipopolysaccharide (LPS)-induced diarrheagenic activity, GI transit, and intestinal fluid content in mice. These investigators found that LPS accumulated abundant fluid in the small intestine in a dose-dependent manner, induced diarrhea, but decreased GI transit. It was observed that pretreatment with BLf significantly attenuated the effects of LPS on diarrheagenic activity and intestinal content, but reversed GI transit time, when compared with NG-nitro-l-arginine-methyl ester (L-NAME, a non-selective NOS inhibitor) or indomethacin (an inhibitor of prostaglandin synthesis). In addition, both plasma NO and PGE_2_ in enterocytes were found to increase in LPS-treated mice and were reversed by BLf. It was concluded that the action of BLf against LPS was specific and that it exerts antidiarrheal and anti-inflammatory activities by modulating the cyclooxygenase pathway (NO and PGE_2_) in the gut [181]. As ovotransferrin and lactoferrin have similar antimicrobial actions, it is possible that ovotransferrin may have similar modes of action as lactoferrin for potential antidiarrheal and anti-inflammatory activities.

In an in vitro study, the turnover rates of intestinal epithelial cells (enterocytes) are governed by the pace of crypt cell proliferation and villus/surface cell shedding [155]. In vitro studies have shown that tight junctional proteins are present at the base of basolateral membranes between two neighboring enterocytes flanking the extruding cells, and thus barrier functions are sustained at the villus tips [182]. Excessive epithelial cell death by pathogenic microbes [183,184], metabolic stress [185], nonsteroidal anti-inflammatory drugs (NSAIDS), and acidic and enzymatic agents [186,187] may lead to villous surface denaturation and gut leakiness.

In essence, the use of microbiota manipulation to improve animal health is a promising and ongoing field of research [141,188]. Nutraceuticals are commonly used to improve gut dysbiosis, but their bioavailability is often very poor. ITPGS has been shown to facilitate the maintenance of a healthy microbiota and microbiome in the GIT in rodents, and therefore may enhance the absorption of nutraceuticals [189].

## 9. Immunomodulatory Properties

Intestinal epithelium maintains a physical barrier to prevent microbial invasion while concomitantly modulating an immune component, which activates host defenses to eliminate invading pathogens [108,176]. These barrier and immune functions of the intestinal epithelium coexist with their equally important mission of maintaining an optimal environment for absorption of nutrients and maintenance of electrolyte balance.

In vitro and in vivo studies demonstrate that normal microbiota exerts profound effects on the mucosal immune system in the gut and regulates immune responses outside the gut [99,100,150,190,191,192]. Mazmanian et al. [193] provided the molecular basis for host-bacterial symbiosis and revealed the archetypal molecule of commensal bacteria (for example, polysaccharide from *Bacteroides fragilis*) that mediates development of the host immune system. The microbiota plays a pivotal role in regulating immunological tolerance to allergen exposure outside the GIT. IT molecules, of which ovotransferrin is the donor of iron for the ITPGS, have a wide range of biofunctions, and are a major component of the mammalian innate immune system.

## 10. ITPGS in Enteric Diseases

Porcine epidemic diarrhea virus (PEDV) is a corona virus, which causes an enteric disease only in pigs. It produces acute, severe diarrhea that transmits rapidly to pigs of all ages. Our unpublished data from an in vivo study show that ovotransferrin (IT) is also effective in controlling diarrhea caused by PEDV. Different groups of piglets were treated with electrolytes and IT. There was complete protection against mortality in IT-treated groups. Data showed that electrolytes and IT, in combination, were potent enough to control severe diarrhea.

Avian coccidiosis is an infectious protozoan disease caused by *Eimeria*. It is one of the most important diseases of poultry, which causes tremendous loss to the industry throughout the world. These protozoa can infect and multiply within the mucosal epithelia in different parts of the gut via oral ingestion. As a result, they cause inflammation, hemorrhage, and diarrhea, and may lead to death. Thus, morbidity and mortality in poultry cost the poultry industry a global loss of about 2.4 billion US dollars annually. Seventy percent of the estimated cost is due to subclinical coccidiosis, as it impacts weight gain and feed conversion ratio (FCR). Recent unpublished data of an in vivo study show that IT can prevent *E. tenella* infestation in 7–21 days old chicks. In addition, weight gain was significantly increased in IT-treated groups. Thus, IT can be used to treat and control subclinical coccidiosis to prevent morbidity and mortality in the poultry industry.

## 11. Concluding Remarks and Future Perspective

Improving gut health is a sound strategy for improving the overall health of an animal. Any new method to do this will be an improvement in the art; this novel ITPGS strategy is one such development. Iron transport polyethylene glycol succinate (ITPGS) is a unique formulation consisting of ovotransferrin from a natural source (eggs) and an α-tocopherol format. The ability of the iron binding and tocopheryl polyethylene glycol succinate appear to exert complimentary actions in the body. ITPGS may serve as a multifunction molecule that can be a solubilizer, stabilizer, surfactant, additive, emulsifier, absorption enhancer, and permeation enhancer. It can offer sustained and targeted delivery of pharmaceuticals, nutraceuticals, and nanomedicines. ITPGS exerts antioxidative, anti-inflammatory, immunomodulatory, antimicrobial and many other biological and pharmacological actions. The ITPGS formulation may prove to be a superior biomaterial for enhancing the efficacy of micelles, nanoparticles, liposomes, or other drug delivery systems and this may have hope for the future. Studies have already shown that ITPGS formulation is efficacious and safe in clinical settings for treating enteric diseases via antimicrobial and antioxidant activities. As microbial imbalances can be caused by several factors resulting in comorbidity situations in the gut, the use of ITPGS may be of great use, especially if polypharmacy approaches are used to treat a patient. This would potentially be due to less total drugs being used in a patient and the chance of more cross drug interactions or toxicities could be minimized with the IPTGS method. The influence of commensal microbiota and its associated medical comorbidities are only beginning to be uncovered. However, it is clear that multi-morbidity and the associated use of multiple medicines is common in older populations [194]. The use of ITPGS methodology when polypharmacy [195,196,197,198] approaches are used may have real value in aging populations of both humans and animals. While further research needs to be conducted for its application in these comorbidity and polypharmacy conditions, the possibility that this strategy can help maintain or improve gut health, and thus improve the overall health of animals, seems very hopeful.
